# *Pediococcus pentosaceus*, a future additive or probiotic candidate

**DOI:** 10.1186/s12934-021-01537-y

**Published:** 2021-02-16

**Authors:** Shiman Jiang, Lingzhi Cai, Longxian Lv, Lanjuan Li

**Affiliations:** 1 State Key Laboratory for Diagnosis and Treatment of Infectious Diseases, National Clinical Research Center for Infectious Diseases and Collaborative Innovation Center for Diagnosis and Treatment of Infectious Diseases, the First Affiliated Hospital, College of Medicine, Zhejiang University, Hangzhou, China; 2 The Infectious Diseases Department, The First People’s Hospital of Wenling, The Affiliated Wenling Hospital of Wenzhou Medical University, Taizhou, China

**Keywords:** *Pediococcus pentosaceus*, Bacteriocin, Probiotics, Food additives, Biopreservative

## Abstract

**Background:**

*Pediococcus pentosaceus*, a promising strain of lactic acid bacteria (LAB), is gradually attracting attention, leading to a rapid increase in experimental research. Due to increased demand for practical applications of microbes, the functional and harmless *P. pentosaceus* might be a worthwhile LAB strain for both the food industry and biological applications.

**Results:**

As an additive, *P. pentosaceus* improves the taste and nutrition of food, as well as the storage of animal products. Moreover, the antimicrobial abilities of *Pediococcus* strains are being highlighted. Evidence suggests that bacteriocins or bacteriocin-like substances (BLISs) produced by *P. pentosaceus* play effective antibacterial roles in the microbial ecosystem. In addition, various strains of *P. pentosaceus* have been highlighted for probiotic use due to their anti-inflammation, anticancer, antioxidant, detoxification, and lipid-lowering abilities.

**Conclusions:**

Therefore, it is necessary to continue studying *P. pentosaceus* for further use. Thorough study of several *P. pentosaceus* strains should clarify the benefits and drawbacks in the future.

## Background

Screening useful bacteria from complex microbial communities is becoming increasingly commonplace. There are billions of microorganisms in the world, but few of them have been tested to determine their features and applications. Among them, the microorganisms called probiotics have already received much publicity for their beneficial effects on humans, animals, plants and foods. In fact, there are not enough probiotics on the market or in clinical practice, which has prompted us to seek insights into other novel bacteria that are both functional and harmless. Currently, only a limited number of probiotics are used for daily maintenance of the intestinal microecological balance and in clinical treatment. Among them, lactic acid bacteria (LAB) have proven worthy of exploration. LAB play important roles in food manufacturing and storage as well as human health promotion [1]. LAB were first recorded in 1907 and used as starter cultures, and they have long been applied as food additives and health promoters [2]. It has already been shown that certain kinds of probiotics can boost disease treatment and improve homeostasis. Both the gut and nonintestinal organs are modulated by probiotics [3]. In addition to *Lactobacillus* and *Bifidobacterium* species, which are the most widely used, *Clostridium* species have recently been proven to possess potent probiotic characteristics [4]. However, these strains are not sufficient for the various demands of humans and industry [5]. Therefore, additional types of bacteria need to be discovered to enrich the application spectrum of probiotics and their applications in food and agriculture.

**Fig. 1:**
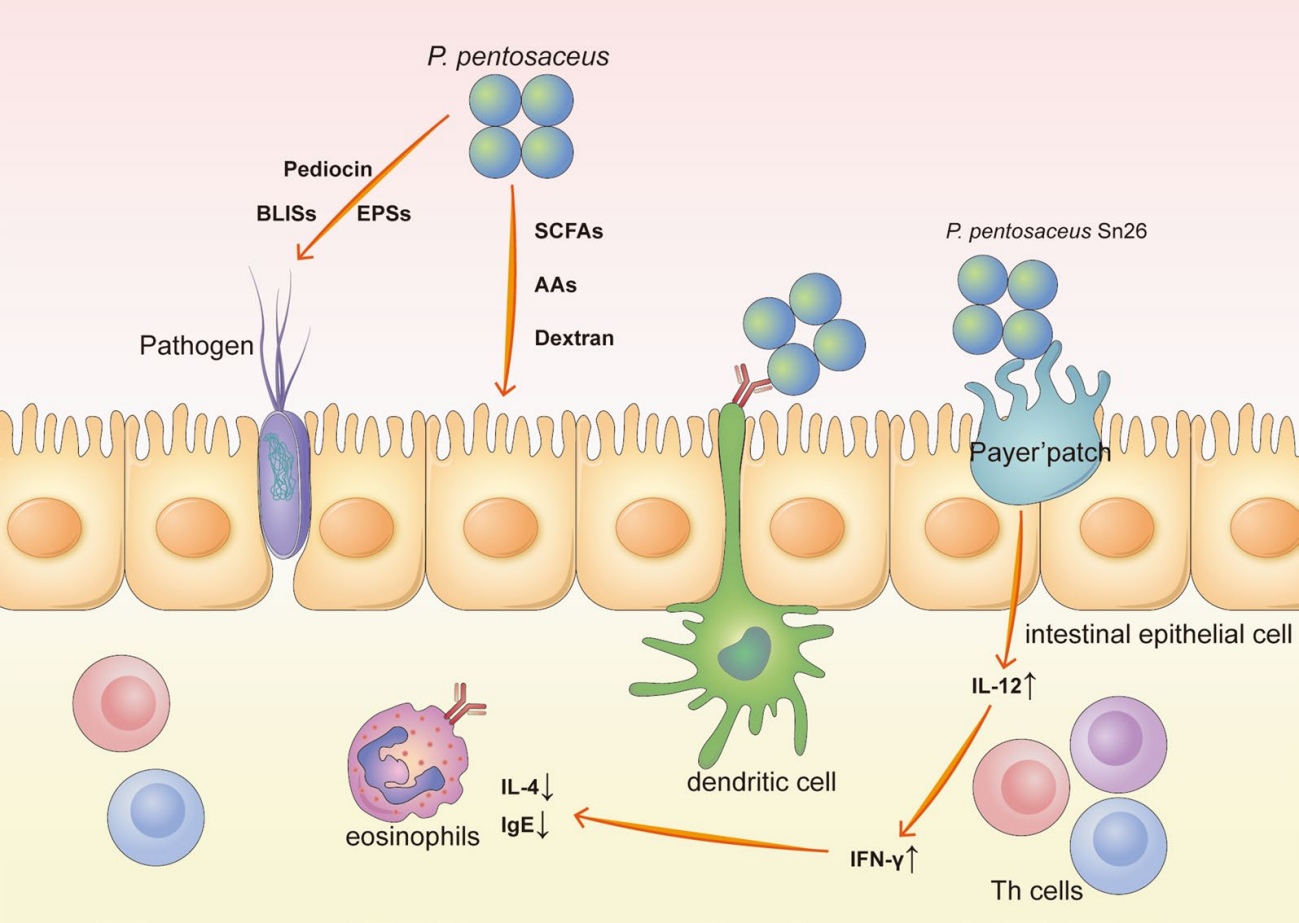
Some *Pediococcus pentosaceus* pathways involved in functional compound production

*Pediococcus pentosaceus*, one type of LAB, has played an increasingly pivotal role in LAB applications in recent years. Isolated from fermented food, aquatic products, raw animal, plant products and faeces, many strains of *P. pentosaceus* were finally proven to have links of the human gastrointestinal tract (GIT) [6]. To date, there is increasing experimental evidence indicating that *P. pentosaceus* may be usable as a biopreservative for foods, plants or animals or as an emerging possible probiotic candidate, as shown in Table 1. *P. pentosaceus* is a cocci-shaped, gram-positive, nonmotile and homofermentative LAB with facultative anaerobic and carbohydrate degradation features [7]. It was already proven in the 1990s that some *P. pentosaceus* strains can be applied in fermentation, as an animal growth biopromoter and as a probiotic [8]. However, most of the properties of *P. pentosaceus* were not well studied at that time. Furthermore, after more than two decades of investigation of *P. pentosaceus*, many other previously undiscovered features have been studied. To date, the problems associated with the practical use of *P. pentosaceus* as a probiotic remain unsolved, for example, there is a lack of knowledge regarding mechanisms, side effects, usage and dosage. There is growing evidence that *P. pentosaceus* and its bacteriocins perform well in both the food industry and intestinal health. Some strains of *P. pentosaceus* produce several functional compounds for different uses, as illustrated in the Fig. 1. Moreover, genomic sequencing verified the ability of both food-derived and animal-derived bacteria to metabolize carbohydrates and horizontally transfer prophage DNA and bacteriocins, etc. [9].

**Table 1:** Summary of studies on *Pediococcus pentosaceus*

The purpose	The strain used	The main findings	References
Improve the taste of food	*P. pentosaceus* T1, *P. pentosaceus* CRAG3, *P. pentosaceus* OA1 and S3N3	Improving texture, sourness and other organoleptic properties	[10–15]
Improve the growth abilities of plants and animals	*P. pentosaceus* Q6, *P. pentosaceus* 4012, *P. pentosaceus* SL001, *P. pentosaceus* PP8	Promoting biological growth and storage	[16–20]
Inhibit bacteria and fungi without bacteriocins or BLISs	*P. pentosaceus* T1, *P. pentosaceus* Z13P, *P. pentosaceus* BaltBio02, *P. pentosaceus* SK25, *P. pentosaceus* -DPS, *P. pentosaceus* L1, *P. pentosaceus* 4I1, *P. pentosaceus* MP12, *P. pentosaceus* TG2, *P. pentosaceus* No. 183, *P. pentosaceus* KCC-23, *P. pentosaceus* PPCS, *P. pentosaceus* HM, *P. pentosaceus* L006	Fighting against bacteria or fungus directly or with EPS, especially L. monocytogenes	[12, 21–27]
Inhibition of inflammation	*P. pentosaceus* AK-23, *P. pentosaceus* ON89A, *P. pentosaceus* KFT18, *P. pentosaceus* NB-17, *P. pentosaceus* Sn26	Exerting anti-inflammatory effects by regulating cytokines and boosting immunity	[28–34]
Antagonism of cancer	*P. pentosaceus* M41, *P. pentosaceus* SL4(PP), *P. pentosaceus* CRAG3, *P. pentosaceus* FP3	Inhibiting the proliferation of cancer, especially CRC	[22, 35–37]
Antioxidant	*P. pentosaceus* Be1, *P. pentosaceus* S-SU6, *P. pentosaceus* MYU 759, *P. pentosaceus* AR243, *P. pentosaceus* KCC-23, *P. pentosaceus* AOA2017, *P. pentosaceus* DK1	Exerting antioxidant effects by scavenging DPPH free radical, O2-, and so on	[38–43]
Reduction of lipids	*P. pentosaceus* LAB6, *P. pentosaceus* LP28, *P. pentosaceus* KID7	Affecting cholesterol metabolism and ameliorating some related diseases	[44–48]
Antidotal action	*P. pentosaceus* FB145 and FB181, *P. pentosaceus* xy46	Degrading or antagonizing toxic substances, such as Cd, ZEA, etc	[49–51]
Utilization of minerals and nutrients	*P. pentosaceus* B3, B11, *P. pentosaceus* CFR R38, R35, *P. pentosaceus* GY23, *P. pentosaceus* UP-2, *P. pentosaceus* TL-3, *P. pentosaceus* HN8	Promoting the biotransformation and utilization of nutrients and minerals, especially phytate	[44, 52–58]
Vaginal protection	*P. pentosaceus* SB83	Surviving manmade vaginal gel and producing antibacterial bacteriocin	[58, 59]

In this review, we have summarized a majority of the studies on *P. pentosaceus* and explored the possibility of its further development as biopreservatives or probiotics. We assume that *P. pentosaceus* has a great impact on the health of animals and especially humans.

## Evidence of *P. pentosaceus* use in food engineering, agriculture and animal husbandry

Different strains of *P. pentosaceus* were detected in foods, plants and animals, acting as flavour enhancers, storage agents or growth stimulators. Some species of bacteria have been gradually shown to provide an array of flavouring agents and food additives in recent years [60]. As food supplements defined by European Food Safety Authority (EFSA), these bacteria play important roles in nutrition supply and physiological functions maintain. Furthermore, members of the *Pediococcus* genus were shown to lack known antibiotic resistance genes [61]. Both traditional and industrial foods are supplemented with particular bacteria to improve taste, nutrition and food safety. Fermentation is an important process to transform bioengineered food to safe, healthy, and green products. In the process of fermentation, *P. pentosaceus* greatly increased the concentration of nitriles and alcohols in Suancai, a Chinese pickled vegetable [62]. Isolated from fermented seeds of *Chrysophyllum albidum* Linn (African star apple), *P. pentosaceus* (ProbtA2b), as a beneficial LAB, changed the physicochemical properties of the fermented food [63].

Notably, *P. pentosaceus* acts as a food additive, providing improved flavour by elevating the concentration of short-chain fatty acids (SCFAs) [10] and the protein hydrolysis of meat [11]. Jang et al. [12] found that *P. pentosaceus* T1 improved texture, sourness and other organoleptic senses and thus could be a starter for original kimchi. Through suppressing LAB, *P. pentosaceus* T1 delayed the maturation of kimchi to improve its utility. *P. pentosaceus* CRAG3 (GenBank accession number JX679020) was first shown to have glucansucrase-producing and prebiotic-utilizing abilities. Then, its high glucansucrase output was certified for contributing to the flavour of dairy products [13, 14]. *P. pentosaceus* OA1 and S3N3 stand out among 41 candidate strains for their superior acidification abilities and proteolytic roles in whole wheat dough fermentation [15]. Gong et al. [64] showed that *P. pentosaceus* could ferment a large number of meats and vegetables, especially Chinese Laomian, and recently, kefir grain-derived *P. pentosaceus* SP2 was proven to be a new candidate for bread fermentation due to its acidity and resistance to mold and rope spoilage [65]. Isolated from traditional sour meat, *P. pentosaceus* SWU73571 and *Lactobacillus curvatus* LAB26, when combined as double-starter culture, improved the flavour, quality and safety of the sour meat [66].

Moreover, in the fields of agriculture and animal husbandry, some studies found that *P. pentosaceus* improved the characteristics and growth abilities of animals and plants. As a major advance in the application of animal husbandry, *P. pentosaceus* DSM 16,244 was certified as safe additive for all animal species by the Panel on Additives and Products or Substances used in Animal Feed (FEEDAP) [67]. With regard to crops, *Elymus nutans*-derived *P. pentosaceus* Q6 was beneficial for the storage quality of *Elymus nutans* silages at low temperature [16]. Thus, *P. pentosaceus* Q6 might be a cold-resistant candidate for high-quality silage storage. With regard to animals, cobia intestine-derived *P. pentosaceus* 4012 was found to act against *Photobacterium damselae* subsp. *Piscicida* (*Pdp*) and hasten cobia growth. In a subsequent experiment, the immune-enhancer *P. pentosaceus* 4012 eliminated vibriosis; this strain was isolated from cobia intestine, making it a promising and profitable strain for use in orange-spotted grouper [17, 18]. *P. pentosaceus* SL001, isolated from many soil samples, not only greatly increased the expression of complement 3 and immunoglobin M but also ameliorated damage to the intestinal villi and goblet cells, indicating promotion of the immune system of grass carp. Its broad-spectrum antibacterial properties also provided an opportunity for probiotic use in freshwater fish [19]. *P. pentosaceus* was found to be beneficial to mud crabs because of its bacteriostatic, immune-enhancing and growth-promoting virtues [68]. Moreover, the application of *P. pentosaceus* PP8 in whiteleg shrimp had the same outcomes [20]. Treatment with *P. pentosaceus* PP8 derived from the gut of juvenile whiteleg shrimp promoted many functions in whiteleg shrimp, such as digestive enzyme activity, disease resistance, growth and immunity. Isolated from human colostrum, *P. pentosaceus* B49 relieved constipation not only by improving gastrointestinal motility and water and electrolyte absorption but also by promoting neurotransmitter transmission and the growth of beneficial short-chain fatty acid-producing bacteria [69]. Isolated from rumen fluid, *P. pentosaceus* S22, with low carbohydrate tolerance, exhibited good adaptability to the gastric juice environment. Research showed that *P. pentosaceus* S22 greatly improved the quality of fermentation of legume silage in vitro [70]. Eveno et al. [71] proposed that *P. pentosaceus* ICVB491 produced bacteriocins and biofilms with low pathogenic risks and high compatibility. Combined with three other LAB, *P. pentosaceus* LUHS183 is involved in the acidification process of feed, leading to great improvement of the hematology indexes, intestinal microorganism distribution and growth of piglets. All four LAB were obtained from the Lithuanian University of Health Sciences collection (Kaunas, Lithuania) [72].

As confirmed by various experiments, many *P. pentosaceus* strains not only could be applied as biopreservatives for foods, crops and livestock but also could be used to accelerate beneficial physical activities. Adding freshness and flavour to food, *P. pentosaceus* is used in the field of food research to promote the development of food engineering from chemical treatment to biological treatment. The usage of *P. pentosaceus* in agriculture is relatively rare, and this species is used mainly in the storage of crops. These strains increase the storage time for plants. But in animal husbandry, related characteristics of *P. pentosaceus* have been explored much more extensively. Through the action of *P. pentosaceus* on animals themselves, improvement of the growth and pathological state of animals can be achieved, so in a sense, *P. pentosaceus* can be called an animal probiotic. *P. pentosaceus* exhibits several beneficial properties, making it a competitive candidate strain for animal feed additives.

## Antimicrobial properties

### Bacteriostatic capacity

*P. pentosaceus* is a strain of LAB that not only enhances the flavour and preservation of food agents but also inhibits colonization by pathogenic bacteria. Many foodborne pathogens colonize food and multiply to cause food decay and poisoning, such as *Salmonella* [73], *Escherichia coli* [74] and *Listeria* [75]. *P. pentosaceus* behaves as a good antipathogen agent when faced with such harmful bacteria. Several experiments were conducted as described below to show that *P. pentosaceus* exhibits good performance against *L. monocytogenes*. *L. monocytogenes* is one of the most common human pathogens, causing meningitis, septicaemia, and abortion [76]. Ubiquitous *L. monocytogenes* contaminates and reproduces in food, eventually resulting in listeriosis [77]. Several strains of *P. pentosaceus* have been shown to restrict the growth of *Listeria* spp., such as *P. pentosaceus* LJR1 [78] and *P. pentosaceus* ATCC 43,200 [79]. For instance, *P. pentosaceus* T1 showed better antilisterial ability in fish products than a disinfectant and nisin, which are famous for their antibacterial abilities [12]. Yuksekdag and Aslim [21] found that five strains of *Pediococcus* spp. isolated from Turkish sausage suppress *L. monocytogenes* by secreting hydrogen peroxide, especially *P. pentosaceus* Z13P. In addition to its high anti-*Listeria* activity, *P. pentosaceus* was found to be effective against other special pathogens, such as *Salmonella* Typhimurium [80] and *Streptococcus salivarius* [81]. Beyond its role in promoting milk yield, *P. pentosaceus* BaltBio02 showed promising antimicrobial activity against *Staphylococcus aureus* and *Pseudomonas aeruginosa* [22]. The traditional Chinese pickle-derived *P. pentosaceus* SK25 produces a large amount of 3-phenyllactic acid, which results in broad-spectrum antibacterial properties, including against a variety of food-spoiling bacteria and fungi [23]. In fact, several strains of *P. pentosaceus* have been shown to have antimicrobial activities for further application [82, 83]. Faba bean contains 8 strains of *P. pentosaceus* that effectively fight against *L. monocytogenes* and *E. coli* [84].

In addition to bacteriocins and bacteriocin-like inhibitory substances (BLISs), exopolysaccharides (EPSs) were confirmed as important metabolites with special functions secreted by *P. pentosaceus*. The EPSs of *P. pentosaceus*-DPS are thermostable and destroy harmful biofilms. *P. pentosaceus*-DPS survived in the human GIT, fighting against *L. monocytogenes* and *E. coli*, and its thermotolerant EPSs destroyed harmful pathogenic bacteria-derived biofilms by inhibiting adhesion [24]. The supernatant of *P. pentosaceus* 4I1, derived from freshwater fish, was effective against foodborne pathogens, such as *E. coli* O157:H7 and *Staphylococcus aureus* KCTC-1621 [26]. *P. pentosaceus* MP12 was obtained from pickled vegetables for inhibiting *Salmonella* invasion to extend the shelf life of pickles [27]. In addition to adhering to the intestinal epithelium, other factors contribute to preventing *Salmonella* invasion.

### Antifungal ability

*P. pentosaceus*, one of the major fungus-inhibiting LAB, behaved well in research [85]. Fungi and their mycotoxins are hazards to the health of their parasitifers and food security. In addition to activity against pernicious bacteria, some *P. pentosaceus* even have antifungal abilities, including *P. pentosaceus* TG2 (collected from Silent Valley National Park) [86], *P. pentosaceus* No. 183 (collected from a spontaneous rye sourdough) [87], and *P. pentosaceus* KCC-23 (collected from Italian ryegrass) [42]. *P. pentosaceus* was found to fight against several common fungi, especially *Aspergillus*, *Penicillium*, *Fusarium* and *Candida albicans*. For instance, *P. pentosaceus* PPCS originated from an Indian traditional fermented dairy product, and the cell-free supernatant (CFS) of *P. pentosaceus* PPCS has the ability to suppress zearalenone (ZEA) activity and inhibit *Fusarium graminearum* [88]. The CFS of *P. pentosaceus* HM (collected from Al-Maray honey) shows fungicidal activity against *Candida* species [89]. The secreted factors of maize leaf-derived *P. pentosaceus* L006 have an impact on fumonisin-producing fungi [90].

### Bacteriocins or BLISs

To date, only one bacteriocin has been applied in food biopreservative use and approved for clinical use by the Food and Drug Administration (FDA): nisin, which is produced by *Lactococcus* [91]. However, more bacteriocins should be identified for use in food supplements and clinical applications. Recently, an increasing number of studies have shown that *P. pentosaceus* antagonizes pathogens through secreting bacteriocins or BLISs [92]. In 2017, Porto et al. [93] provided a detailed overview of the cultivation and mechanisms of *Pediococcus* spp. and their pediocins [93]. Bacteriocins, polypeptides synthesized by ribosomes, have antibacterial properties, especially against foodborne pathogens. The bacteriocins of class II-a consist of pediocin-like single peptides. Bacteriocins from *Pediococcus* spp. are small and heat-stable peptides known as pediocins and have hydrophobic and cationic properties [92, 94]. The pediocins known so far are listed in Table 2. The bacteriocin of *P. pentosaceus* exhibits bactericidal ability against pathogens, such as *L. monocytogenes* and *Clostridium perfringens* [95]. By screening thousands of bacteria from kimchi, *P. pentosaceus* K 23–2 and its pediocin K 23–2 were found to inhibit various gram-positive pathogens [96]. *P. pentosaceus* LMQS 331.3, isolated from traditionally fermented German sausages, produces a bacteriocin with good stability and effective suppression of *Listeria* spp. [97]. In addition to antagonistic activity against pathogens, *P. pentosaceus* CFF4, as an isolate from different food matrices, was found to adhere to Caco-2 cells [98]. Fermented triticale silage-derived *P. pentosaceus* (TC48) not only produced better-quality silage but also inhibited *Pseudomonas aeruginosa*, *Enterococcus faecalis*, *S. aureus* and *E. coli* [99]. *P. pentosaceus* ST65ACC, a poorly cytotoxic coagulin A producer from raw milk cheese, survived in the GIT and coaggregated with *L. monocytogenes* for pathogen elimination [100–102]. A strain of *P. pentosaceus* was shown to perform well in many aspects. *P. pentosaceus* LJR1, an isolate from goat rumen fluid, secretes a 4.6 kDa bacteriocin called bacteriocin LJR1. When added to whiteleg shrimp, bacteriocin LJR1 inhibited *Listeria monocytogenes* [78]. Additionally, idly batter-derived *P. pentosaceus* VJ13 produced a bacteriocin called bacteriocin VJ13 with high thermal and chemical stabilities to fight against human pathogens, especially *L. monocytogenes* [103]. Expressed in human breast milk and successfully passed through the upper GIT, *P. pentosaceus* OZF secretes certain bacteriocins with anti-*Listeria* activity [104]. Bacteriocin ST44AM produced by marula-derived *P. pentosaceus* ST44AM is a class II-a pediocin-like peptide. In addition to its antimicrobial activity, this pediocin PA-1 deuterogenic bacteriocin has a synergistic effect with ciprofloxacin [105]. Penocin A, the bacteriocin of *P. pentosaceus* ATCC 25,745, showed a broad spectrum of bacterial inhibition, such as against *Clostridium* and *Listeria* species. The genome of *P. pentosaceus* ATCC 25,745, which was isolated from plants, is already known [106, 107]. The *pen*A gene was verified to promote pediocin production [108]. Moreover, both pentocin L and pentocin S were able to inhibit an extensive spectrum of pathogens, especially *Clostridium sporogenes* ATCC 11,259 [109]. *P. pentosaceus* DT016 weakened the proliferation of *L. monocytogenes* by pediocin DT016 in vegetable storages. In short, bacteriocins produced by *P. pentosaceus* showed good antibacterial performance, but their food safety needs to be evaluated for further clinical and food application.

**Table 2:** Bacteriocins produced by *Pediococcus pentosaceus*

Bacteriocin	Producing organism	Source	Classification	Reference
Bacteriocin LJR1	*P. pentosaceus* LJR1	Goat rumen liquor	4.6 kDa	[78]
Pediocin GS4	*P. pentosaceus* GS4	Indian fermented food (Khadi)	Class II-a	[94]
Pediocin MZF16	*P. pentosaceus* MZF16	Artisanal Tunisian meat, dried Ossban	Class II-a	[110]
Bacteriocin LB44	*P. pentosaceus* LB44	Dairy soil	∼6 kDa	[111]
Coagulin A	*P. pentosaceus* ST65ACC	Raw milk cheese	Class II-a	[102]
Bacteriocin VJ13B	*P. pentosaceus* VJ13B	Idly batter	Class II-a	[103]
Bacteriocin (OZF)	*P. pentosaceus* OZF	Human breast milk	-	[104]
Bacteriocin ST44AM	*P. pentosaceus* ST44AM	Marula	Class II-a	[105]
Pediocin K23-2	*P. pentosaceus* K23-2	Kimchi	~5 kDa	[96]
Penocin A	*P. pentosaceus* ATCC 25745	Plant	4684.6 Da	[108]
Pediocin PA-1 (AcH)	Multiple LAB (pS34 from *P. pentosaceus* S34)	Food products	Class II-a	[112]
Pediocin ACCEL	*P. pentosaceus* ACCEL	Vacuum-packaged meat	Class II-a	[113]
Pentocin L	*P. pentosaceus* L	Refrigerated pork	27 kDa	[109]
Pentocin S	*P. pentosaceus* S	Refrigerated pork	25 kDa	[109]
Pediocin A	*P. pentosaceus* FBB61 (ATCC 43200)	Fermented cucumbers	80 kDa	[114]
Pediocin N5p	*P. pentosaceus* N5p	Wine	-	[115]

Similar to bacteriocins, BLISs, a previously unidentified category of molecules, lead to bacteria death through depolarized membrane and cytoplasmic content release. *P. pentosaceus* ATCC 43,200, also known as *P. pentosaceus* FBB61, was first isolated from fermented cucumbers in 1953 [116], and further studies were carried out. The BLIS pediocin A is most active at pH 5.0 with no additional sugar supplements in the medium [114, 117]. Then, it was determined that inulin and sucrose accelerate the production of pediocin A [92]. However, it was proven that a *P. pentosaceus* BLIS produced by *P. pentosaceus* ATCC 43,200 not only inhibits the growth of *Listeria seeligeri* but also improves the appearance of pork ham. Moreover, the BLIS may have better antibacterial effects than Nisaplin [79, 118]. Furthermore, the secretion of BLISs by *P. pentosaceus* is influenced by many factors, such as pH and temperature. Gutierrez-Cortes et al. [119] designed an experiment to elucidate pediocin upregulation during coculture with *Lactobacillus*. In summary, both bacteriocin and BLIS produced by *P. pentosaceus* were competent substitutes for antimicrobial agents. As a strain of bacteriocinogenic LAB, *P. pentosaceus* has the ability to inhibit a wide variety of pathogens.

## Probiotic applications

Until now, basic and clinical research on probiotics has progressed rapidly, but probiotics still have not been applied as drugs worldwide [120]. In the United States, probiotics have been recommended by clinical doctors as evidence-based conventional or medical foods and dietary supplements under the guidance of the US Food and Drug Administration (FDA). Many probiotic strains are considered Generally Recognized as Safe (GRAS) to use in foods (https://www.fda.gov/animal-veterinary/animal-food-feeds/generally-recognized-safe-gras-notification-program). In addition, the European Food Safety Authority (EFSA) evaluated a number of traditional species of probiotics as safe for application in food by the Qualified Presumption of Safety (QPS) [121]. From October 2019 to March 2020, EFSA found that only 6 microorganisms met the standard from 39 notifications [122].

The interactions between *P. pentosaceus* and the gut microbiota alone have been widely analysed, but not many studies have evaluated them by -omics approaches, such as transcriptomics, proteomics, metagenomics, and 16S rDNA/rRNA gene sequencing. For example, gastrointestinal peristalsis was partially improved by treatment with *P. pentosaceus* B49, as determined by analysing the transcriptomic outcome in the colon [69]. The cell adherence function of *P. pentosaceus* GS4 was proved to produce a 98 kDa surface layer protein, as determined by proteomic technology such as SDS-PAGE and size exclusion chromatography [123]. Through metagenomic sequencing, *P. pentosaceus* strains were selected from hundreds of LAB for their beneficial characteristics. For instance, *P. pentosaceus* strains were found in a dominant position in wheat fermentation, in addition to *Lactobacillus plantarum* and *Lactobacillus fermentum* [124]. The most widely used method is 16S rDNA/rRNA gene sequence analysis, which identified *P. pentosaceus* among a variety of bacteria. Hundreds of *P. pentosaceus* strains were selected by screening various samples and further identified based on the results of 16S rRNA gene sequencing [125]. Through -omics approaches, additional features and functions of *P. pentosaceus* were determined for further probiotic application.

### Anti-inflammatory ability

*P. pentosaceus* was verified to sustain internal environmental homeostasis, for example, by enforcing systemic immunity and enhancing anti-inflammatory ability. *P. pentosaceus* showed its anti-inflammatory abilities in the host through upregulating or downregulating lipopolysaccharides (LPS) or cytokines. Many studies were conducted to examine the relationship between inflammatory response and *P. pentosaceus,* as described below. High burdens of gram-negative pathogens give rise to intestinal health disorders because of their LPS. The heat shock protein (HSP) of pickle-derived *P. pentosaceus* AK-23 is useful for neutralizing LPS, which is finally degraded to polysaccharides and fatty acids [28]. Onion-derived *P. pentosaceus* ON89A improves the anti-inflammatory effect of the Chinese herb *Cordyceps militaris* (*C. militaris*) via the GRC-ON89A hexane fraction (GRC-ON89A-Hex). LPS-stimulated RAW 264.7 macrophages were affected by GRC-ON89A-Hex, resulting in decreases in TNF-α, cyclooxygenase 2 (COX2) and inducible NO synthase (iNOS). Enterotoxigenic *E. coli* F4^+^ antagonism by *P. pentosaceus* L1 showed anti-inflammatory effects by downregulating the levels of interleukin (IL)-6, tumour necrosis factor (TNF)-α and IL-8 [25]. Furthermore, oral administration of fermented *C. militaris* with *P. pentosaceus* ON89A in cyclophosphamide (CY)-induced mice improved its immunostimulatory abilities and obtain more nutrition acquisition [29, 30]. Moreover, the PE-EPS of [31] kimchi-derived *P. pentosaceus* KFT18 was administered to CY-mediated immunosuppressed mice, which mediated spleen and thymus development and lymphocyte and cytokine production. *P. pentosaceus* KFT18, which could even act as an immunomodulator, stimulated a protective immune response [32]. In vitro, pickled vegetable-isolated *P. pentosaceus* NB-17 was applied to ovalbumin (OVA)-sensitized mouse spleen cells. The improvement in IL-12 and interferon (IFN)-γ levels showed a possibility for improved immunity [33]. In the model of OVA-treated diarrheic mice, *P. pentosaceus* Sn26 also downregulated OVA-specific IgE levels in serum and upregulated IL-12 and IFN-γ levels. Maintaining the Th1/Th2 balance, the Japanese Sunki pickle-derived *P. pentosaceus* Sn26 is good for modulating type 1 allergic reactions [34].

### Anticarcinogenic properties

Several cancers, especially colorectal cancer (CRC), were found to be ameliorated by certain kinds of probiotics. Also, some experiments were conducted to determine the anticancer functions of *P. pentosaceus*. As a new probiotic candidate, *P. pentosaceus* might stand out from a range of already known probiotics. *P. pentosaceus* M41, derived from a marine source, excreted EPS-M41 with antitumor and antioxidant activities, including high anti-Caco-2 and anti-MCF-7 efficacy [35]. Protein p8 is a therapeutic substance that is certified to decrease the mass of CRC and enhance antiproliferative activity. Lele et al. [22] invented p8-secreting induction kit targets based on *P. pentosaceus* SL4 (PP). Recombinant *P. pentosaceus* SL4 offers an alternative means of delivering p8 for oncotherapy. Dextran, a product of *P. pentosaceus* CRAG3, inhibited colon cancer (HT29) and cervical cancer (HeLa) cell lines. Extracted from fermented cucumber, *P. pentosaceus* CRAG3 is a novel probiotic candidate with anticancer functions [36]. Among 81 isolates from infant faeces, *P. pentosaceus* FP3 inhibited the proliferation of colon cancer and induced the production of SCFAs [37]. This study proved that *P. pentosaceus* FP3 might serve as a prophylactic or therapeutic approach for colon cancer. As mentioned above, some strains of *P. pentosaceus* may have a brighter future.

### Antioxidant applications

The antioxidant capacity of LAB includes the scavenging capacities of α,α-diphenyl-β-picrylhydrazyl (DPPH) free radical, O_2_^−^, and so on. Through these abilities, some related diseases, such as neurodegenerative disease, cardiovascular disease and, more importantly, senility, benefit greatly. Some strains of *P. pentosaceus* are adept at scavenging hydrogen peroxide, such as *P. pentosaceus* Be1 (collected from fermented food) [38] and *P. pentosaceus* S-SU6 (collected from the gut of blue mackerel) [39]. *P. pentosaceus* MYU 759 was found to have hydroxyl radical antioxidant capacity (HORAC) due to the secretion of acidic EPS, which is highly beneficial in its isolation from soymilk yogurt as an antioxidant product [40]. Additionally, *P. pentosaceus* AR243, an isolated LAB from Chinese fermented foods, had significant scavenging abilities for hydroxyl radicals and DPPH free radicals, resulting in further inhibition of lipid peroxidation [41]. *P. pentosaceus* KCC-23 survived in the low-pH environment of gastric juice and not only inhibited fungus and bacteria effectively but also exhibited its own biological potential. Italian ryegrass-derived *P. pentosaceus* KCC-23 was determined to have a strong free radical-scavenging effect and cholesterol-lowering ability [42]. *Eleusine coracana*-derived *P. pentosaceus* AOA2017 amplified the antioxidant ability of Korean Yak-Kong, thereby possibly resulting in atherosclerosis prevention [43]. *P. pentosaceus* DK1 was applied to *Lavandula angustifolia* extract for its antiaging effect. In addition, it turned out that *Diospyros kaki* fruit-derived *P. pentosaceus* DK1 effectively downregulated UVB-mediated MMP-1 expression and collagen [126]. Additionally, a strain of *P. pentosaceus* in Harbin dry sausage possessed the ability to scavenge radicals. It might also be an antioxidant of meat [127].

### Lipid-lowering effects

Recently, probiotic supplementation was shown to decrease cholesterol levels in humans via several mechanisms [47]. *P. pentosaceus* strains were also shown to affect cholesterol metabolism. For instance, Malaysian *P. pentosaceus* LAB6 decreased cholesterol without bile salts by 54% and cholesterol with bile salts by 58% compared to other LAB [44]. Applied to obese mice with high-fat diets, *P. pentosaceus* LP28 resulted in weight reduction and decreased the concentrations of cholesterol and triglycerides. These plant-derived LAB were confirmed as promising antiobesity bacteria for preventing metabolic syndrome [45, 46]. Both in vitro and in vivo experiments were carried out, and *P. pentosaceus* KID7 survived in the gastrointestinal environment and lowered cholesterol levels. Oral administration of KID7 in atherogenic diet-induced mice greatly ameliorated elevated cholesterol levels [47]. Through modulating the gut microbiota, finger millet gruel-derived *P. pentosaceus* KID7 ameliorates the nonalcoholic fatty liver disease (NAFLD) state [48]. As an isolate from Northeast pickled cabbage, *P. pentococcus* PP04 ameliorated blood lipids and markers of liver injury and inflammation in hyperlipidemia model C57BL/6 N mice through the AMPK signaling pathway [128].

### Detoxification

*P. pentosaceus* strains might act as novel potent biological antidotes for reducing or preventing the presence of toxic substances in human bodies. Through physical methods, detoxification abilities have been reported for several strains of *P. pentosaceus*. *P. pentosaceus* not only affected the substance absorption and excretion of the intestine but also upregulated toxic substance decomposition by the detoxification ability of the liver, such as reducing blood ammonia, heavy metal ion, and endotoxin levels [49]. Le and Yang [50] carried out experiments to show that *P. pentosaceus* FB145 and FB181, derived from fermented seafood, were capable of reducing the toxicity of cadmium (Cd). They found that Cd combined with bacterial cells by specific several functional groups and thus decreased Cd bioaccessibility in vitro to 44.7–46.8%. Administration of *P. pentosaceus* xy46 (CCTCC number: M2018352) protected the male reproductive system of mice from the poisonous effects of ZEA at a relatively low dose [51]. The ability to detoxify heavy metal ions and other toxic metabolites gives *P. pentosaceus* a bright future.

### Promotion of mineral and nutrient utilization

*P. pentosaceus* converts food into nutrients and promote mineral utilization. To the best of our knowledge, phytic acid is harmful to mineral bioavailability. Moreover, sourdough-derived *P. pentosaceus* B3 and B11 and chicken intestine-derived *P. pentosaceus* CFR R38 and R35 were able to degrade phytate and promote mineral bioavailability [52, 53]. Similarly, from among 60 strains of LAB from cereal- and pulse-based fermented mixtures, *P. pentosaceus* CFR R123 not only significantly improved the availability of calcium but also reduced cholesterol and β-galactosidase levels. *P. pentosaceus* CFR R123 degraded 43% of phytate in one hour in an experiment designed by Raghavendra et al. [54, 55]. These phytate-degrading *P. pentosaceus* have potential as nutritive food additives [54, 55]. In the model of grass carp sausages, *P. pentosaceus* GY23 showed a proteolytic profile that was different from that of other LAB [56]. With high proteolysis ability, Malaysian food-derived *P. pentosaceus* UP-2 could produce 15 extracellular amino acids (AAs) for body nutrition or biological functions [57]. Isolated from tempeh-fermented soybean cake, *P. pentosaceus* TL-3 was confirmed to have the highest threonine and methionine production among LAB [44]. γ-Aminobutyric acid (GABA) is an amino acid that has been reported to ameliorate diabetes, hypertension, and even cancer development. There are several kinds of LAB that produce GABA, and *P. pentosaceus* HN8, which was collected from Thai fermented pork sausage, is one such LAB [58]. Moreover, one strain of *P. pentosaceus* in Assam produced dextran, which is nonpoisonous and bioactive. It might be a future delivery system material for drugs or bioengineered tissue [129].

### Vaginal delivery connection

Some probiotics are versatile and can be used in the protection of the vagina, especially when giving birth, including *P. pentosaceus*. From a total 35 strains of bacteriocinogenic *P. pentosaceus*, *P. pentosaceus* SB83 was selected for vaginal protection. *P. pentosaceus* SB83 is sensitive to a wide spectrum of antibiotics [58]. Apart from bacteriocin production, *P. pentosaceus* SB83 was tested for survival in glycerol and in manmade vaginal gel [59].

### Dilemma

When applied to the human body, *P. pentosaceus* is definitely also harmful to health if it is not in the correct location. Duchaine et al. [130] found that *P. pentosaceus* produced abrupt inflammation in clinical use, resembling *Saccharopolyspora rectivirgula*. Additionally, the contradiction between the research and development of probiotics and practical use is currently a conundrum. As Suez et al. [131] mentioned, the supervision of the safety, efficacy and cost effectiveness of probiotics is essential to optimize the whole industry. Moreover, the evaluation of all aspects of probiotics has not been fully studied. The risk of probiotics for the human body, in particular, has not been sufficiently evaluated in either scientific research or clinical practice [132].

Overall, even though it is increasingly clear that additional safety assessment is needed for screening and selection of probiotics, *P. pentosaceus* is a preferable choice for probiotics.

## Potential special ability of *P. pentosaceus*

Several strains of *P. pentosaceus* have been studied more deeply than others and achieved special stats as candidate probiotics, such as *P. pentosaceus* ATCC 43,200 and *P. pentosaceus* KID7. For instance, *P. pentosaceus* GS4, a likely future probiotic, had been tested for its basic characteristics, biological processes and effects on the body. Sukumar and Ghosh [133] found that Indian fermented food-derived *P. pentosaceus* GS4 has antibacterial ability, in addition to being equipped with basic antioxidative properties, cholesterol absorption ability, and acid and bile salt tolerance. Furthermore, Dubey et al. [134–136] carried out experiments to determine that *P. pentosaceus* GS4 can become biohydrogenated, that it produces conjugated linolenic acid, that it has reduced toxicity, and that it inhibits mouse colon carcinogenesis. *P. pentosaceus* GS4 also survived under sustained gastric acid irritation and during cold storage. Recently, pediocin GS4 was purified and certified for hard denaturation [94, 137]. Similarly, *P. pentosaceus* MZF16 colonized and improved the intestinal tract without any cytotoxicity, secreting a BLIS named pediocin MZF16 with anti-*Listeria* ability [110]; this strain was collected from artisanal Tunisian meat. Adhering to the surface of the GIT, human breast milk-derived *P. pentosaceus* OZF might be a probiotic because of its antipathogen bacteriocin [104]. Even ginseng root-extracted *P. pentosaceus* HLJG0702 was found to be able to produce HLJG0701. HLJG0701 also significantly improved the concentration of the ginsenosides Rg5/Rk1 to inhibit acetylcholinesterase in scopolamine-induced memory dysfunction mice, which manifested as an improvement in brain function [138].

Moreover, *P. pentosaceus* LI05, identified by screening the faeces of healthy volunteers, has achieved substantial success in recent years. In a D-galactosamine-treated rat model, *P. pentosaceus* LI05 obviously ameliorated liver enzyme levels and the morphology of the terminal ileum and liver. The microflora distribution was altered, and bacterial translocation was reduced upon oral administration of *P. pentosaceus* LI05 [139]. Shi et al. [140] found that *P. pentosaceus* LI05 corrected hepatic fibrosis by ameliorating inflammatory cytokine levels and the intestinal bacterial flora distribution in a CCl_4_-damaged liver cirrhosis model. Furthermore, *P. pentosaceus* LI05 was certified to inhibit *Clostridium difficile* infection (CDI) in mice. By reducing inflammation and upregulating tight junction proteins, *P. pentosaceus* LI05 prevented pathogen colonization of the intestine [141]. In a DSS-induced colitis model, *P. pentosaceus* LI05 improved the status of colitis and remarkably augmented the diversity of the microbiota and the level of SCFAs [142]. These *Pediococcus* spp. showed infinite potential for the maintenance of body health status. Despite counteracting diseases and maintaining health, no side effects of these *Pediococcus* spp. have been discovered yet.

## Technological potential

With regard to converting *P. pentosaceus* to a commercial product, several properties are desired. For instance, the property most worth discussing is the resistance of *P. pentosaceus* to different procedures, such as lyophilization, atomization, salinization, etc. An oro-gastrointestinal transit (OGT) tolerance assay [143] and a Caco-2 cell culture and adhesion assay [144] were performed and showed that *P. pentosaceus* is resistant to gastric acid and can adhere to the intestines. Given its tolerance to the freeze-drying and storage conditions, *P. pentosaceus* KID7 was approved for storage by lyophilization [47]. *P. pentosaceus* CRAG3 has tolerance for bile salts and even the ability to degrade bile salts [13]. Additional features are needed, for example, adhesion ability and bacteriocin‐producing ability. The biofilm formation ability of *P. pentosaceus* has potential industrial applications, such as in drug research and development, as only in biofilm cells of *P. pentosaceus* can proteins related to probiotic properties be expressed and enriched [145]. Much more research is needed to test the possibility of commercial use of *P. pentosaceus*.

## Future direction

Identifying the potential uses and commercial properties of *P. pentosaceus* is the most important next step. As a promising candidate in the world of probiotics and beneficial bacteria, selecting the most promising strain of *P. pentosaceus* is the next most important task, not to mention the preparation of complete bacterial formulations and systematic and complete evaluation of the characteristics and disadvantages of *P. pentosaceus*, which are also necessary. Then, the bacteria can be prepared for direct application in agriculture, animal husbandry, the food industry or clinical settings in the near future.

## Conclusions

*P. pentosaceus*, a potentially predominant probiotic strain in the future, has been studied by many researchers since it was first described in the 1960s. In playing an all-important role in the food industry and animal husbandry, *Pediococcus* spp. acts on the intestines and improves overall body condition. *P. pentosaceus* has high potential to achieve probiotic status.

## Data Availability

Not applicable.

## Abbreviations

LAB: Lactic acid bacteria
BLISs: Bacteriocin-like substances
GIT: Human gastrointestinal tract
EFSA: European Food Safety Authority
FEEDAP: Panel on Additives and Products or Substances used in Animal Feed
SCFAs: Short-chain fatty acids
EPSs: Exopolysaccharides
IL: Interleukin
TNF: Tumor necrosis factor
IFN: Interferon
CFS: Cell-free supernatant
ZEA: Zearalenone
FDA: Food and Drug Administration
LPS: Lipopolysaccharide
HSP: Heat shock protein
COX2: Cyclooxygenase 2
iNOS: Inducible NO synthase
CY: Cyclophosphamide
OVA: Ovalbumin
IFN: Interferon
CRC: Colorectal cancer
DPPH: α,α-Diphenyl-β-picrylhydrazyl
HORAC: Hydroxyl radical antioxidant capacity
NAFLD: Nonalcoholic fatty liver disease
Cd: Cadmium
AAs: Amino acids
GABA: γ-Aminobutyric acid
CDI: *Clostridium difficile* Infection

## References

[1] Gad SA, El-Baky RMA, Ahmed ABF, Gad GFM (2016). In vitro evaluation of probiotic potential of five lactic acid bacteria and their antimicrobial activity against some enteric and food-borne pathogens. Afr J Microbiol Res.

[2] Zommiti M, Feuilloley MGJ, Connil N (2020). Update of probiotics in human world: a nonstop source of benefactions till the end of time. Microorganisms.

[3] Lee ES, Song EJ, Nam YD, Lee SY (2018). Probiotics in human health and disease: from nutribiotics to pharmabiotics. J Microbiol.

[4] Guo P, Zhang K, Ma X, He P (2020). *Clostridium* species as probiotics: potentials and challenges. J Anim Sci Biotechnol.

[5] Min BE, Hwang HG, Lim HG, Jung GY (2017). Optimization of industrial microorganisms: recent advances in synthetic dynamic regulators. J Ind Microbiol Biotechnol.

[6] Barros RR, Carvalho MG, Peralta JM, Facklam RR, Teixeira LM (2001). Phenotypic and genotypic characterization of *Pediococcus* strains isolated from human clinical sources. J Clin Microbiol.

[7] Dobrogosz WJ, Stone RW (1962). Oxidative metabolism in *Pediococcus pentosaceus* II. Factors controlling the formation of oxidative activities. J Bacteriol..

[8] Danielsen M, Simpson PJ, O’Connor EB, Ross RP, Stanton C (2007). Susceptibility of *Pediococcus* spp.. to antimicrobial agents. J Appl Microbiol..

[9] Jiang J, Yang B, Ross RP, Stanton C, Zhao J, Zhang H (2020). Comparative genomics of *Pediococcus pentosaceus* isolated from different niches reveals genetic diversity in carbohydrate metabolism and immune system. Front Microbiol.

[10] Wang Y, Sun J, Zhong H, Li N, Xu H, Zhu Q (2017). Effect of probiotics on the meat flavour and gut microbiota of chicken. Sci Rep.

[11] Sun F, Hu Y, Chen Q, Kong B, Liu Q (2019). Purification and biochemical characteristics of the extracellular protease from *Pediococcus pentosaceus* isolated from Harbin dry sausages. Meat Sci.

[12] Jang S, Lee D, Jang IS, Choi HS, Suh HJ (2015). The culture of *Pediococcus pentosaceus* T1 inhibits *Listeria* proliferation in salmon fillets and controls maturation of kimchi. Food Technol Biotechnol.

[13] Shukla R, Goyal A (2014). Probiotic potential of *Pediococcus pentosaceus* CRAG3: a new isolate from fermented cucumber. Probiotics Antimicrob Proteins.

[14] Shukla R, Goyal A (2014). Purified dextransucrase from *Pediococcus pentosaceus* CRAG3 as food additive. Indian J Exp Biol.

[15] Montemurro M, Celano G, De Angelis M, Gobbetti M, Rizzello CG, Pontonio E (2020). Selection of non-*Lactobacillus* strains to be used as starters for sourdough fermentation. Food Microbiol.

[16] Xu DM, Ke WC, Zhang P, Li FH, Guo XS (2019). Characteristics of *Pediococcus pentosaceus* Q6 isolated from *Elymus nutans* growing on the Tibetan Plateau and its application for silage preparation at low temperature. J Appl Microbiol.

[17] Xing CF, Hu HH, Huang JB, Fang HC, Kai YH, Wu YC (2013). Diet supplementation of *Pediococcus pentosaceus* in cobia (*Rachycentron canadum*) enhances growth rate, respiratory burst and resistance against photobacteriosis. Fish Shellfish Immunol.

[18] Huang JB, Wu YC, Chi SC (2014). Dietary supplementation of *Pediococcus pentosaceus* enhances innate immunity, physiological health and resistance to *Vibrio anguillarum* in orange-spotted grouper (*Epinephelus coioides*). Fish Shellfish Immunol.

[19] Gong L, He H, Li D, Cao L, Khan TA, Li Y (2019). A new isolate of *Pediococcus pentosaceus* (SL001) with antibacterial activity against fish pathogens and potency in facilitating the immunity and growth performance of grass carps. Front Microbiol.

[20] Won S, Hamidoghli A, Choi W, Bae J, Jang WJ, Lee S (2020). Evaluation of potential probiotics *Bacillus subtilis* WB60, *Pediococcus pentosaceus*, and *Lactococcus lactis* on growth performance, immune response, gut histology and immune-related genes in whiteleg shrimp, *Litopenaeus vannamei*. Microorganisms.

[21] Yuksekdag Z, Aslim B (2010). Assessment of potential probiotic- and starter properties of *Pediococcus* spp. isolated from Turkish-type fermented sausages (sucuk). J Microbiol Biotechnol..

[22] Lele V, Zelvyte R, Monkeviciene I, Kantautaite J, Stankevicius R, Ruzauskas M (2019). Milk production and ruminal parameters of dairy cows fed diets containing *Lactobacillus sakei* KTU05-6 and *Pediococcus pentosaceus* BaltBio02. Pol J Vet Sci.

[23] Yu S, Zhou C, Zhang T, Jiang B, Mu W (2015). Short communication: 3-phenyllactic acid production in milk by *Pediococcus pentosaceus* SK25 during laboratory fermentation process. J Dairy Sci.

[24] Abid Y, Casillo A, Gharsallah H, Joulak I, Lanzetta R, Corsaro MM (2018). Production and structural characterization of exopolysaccharides from newly isolated probiotic lactic acid bacteria. Int J Biol Macromol.

[25] Yin H, Ye P, Lei Q, Cheng Y, Yu H, Du J (2020). *In vitro* probiotic properties of *Pediococcus pentosaceus* L1 and its effects on enterotoxigenic *Escherichia coli*-induced inflammatory responses in porcine intestinal epithelial cells. Microb Pathog.

[26] Bajpai VK, Han JH, Rather IA, Park C, Lim J, Paek WK (2016). Characterization and antibacterial potential of lactic acid bacterium *Pediococcus pentosaceus* 4I1 isolated from freshwater fish *Zacco koreanus*. Front Microbiol.

[27] Chiu HH, Tsai CC, Hsih HY, Tsen HY (2008). Screening from pickled vegetables the potential probiotic strains of lactic acid bacteria able to inhibit the *Salmonella* invasion in mice. J Appl Microbiol.

[28] Asami K, Kondo A, Suda Y, Shimoyamada M, Kanauchi M (2017). Neutralization of lipopolysaccharide by heat shock protein in *Pediococcus pentosaceus* AK-23. J Food Sci.

[29] Kwon HK, Jo WR, Park HJ (2018). Immune-enhancing activity of *C. militaris* fermented with *Pediococcus pentosaceus* (GRC-ON89A) in CY-induced immunosuppressed model. BMC Complement Altern Med..

[30] Kwon HK, Song MJ, Lee HJ, Park TS, Kim MI, Park HJ (2018). *Pediococcus pentosaceus*-fermented *Cordyceps militaris* inhibits inflammatory reactions and alleviates contact dermatitis. Int J Mol Sci.

[31] Garcia-Ruiz A, Gonzalez de Llano D, Esteban-Fernandez A, Requena T, Bartolome B, Moreno-Arribas MV (2014). Assessment of probiotic properties in lactic acid bacteria isolated from wine. Food Microbiol..

[32] Shin JS, Jung JY, Lee SG, Shin KS, Rhee YK, Lee MK (2016). Exopolysaccharide fraction from *Pediococcus pentosaceus* KFT18 induces immunostimulatory activity in macrophages and immunosuppressed mice. J Appl Microbiol.

[33] Jonganurakkun B, Wang Q, Xu SH, Tada Y, Minamida K, Yasokawa D (2008). *Pediococcus pentosaceus* NB-17 for probiotic use. J Biosci Bioeng.

[34] Masuda T, Kimura M, Okada S, Yasui H (2010). *Pediococcus pentosaceus* Sn26 inhibits IgE production and the occurrence of ovalbumin-induced allergic diarrhea in mice. Biosci Biotechnol Biochem.

[35] Ayyash M, Abu-Jdayil B, Olaimat A, Esposito G, Itsaranuwat P, Osaili T (2020). Physicochemical, bioactive and rheological properties of an exopolysaccharide produced by a probiotic *Pediococcus pentosaceus* M41. Carbohydr Polym.

[36] Shukla R, Goyal A (2013). Novel dextran from *Pediococcus pentosaceus* CRAG3 isolated from fermented cucumber with anti-cancer properties. Int J Biol Macromol.

[37] Thirabunyanon M, Hongwittayakorn P (2013). Potential probiotic lactic acid bacteria of human origin induce antiproliferation of colon cancer cells via synergic actions in adhesion to cancer cells and short-chain fatty acid bioproduction. Appl Biochem Biotechnol.

[38] Watanabe A, Kaneko C, Hamada Y, Takeda K, Kimata S, Matsumoto T (2016). Isolation of lactic acid bacteria exhibiting high scavenging activity for environmental hydrogen peroxide from fermented foods and its two scavenging enzymes for hydrogen peroxide. J Gen Appl Microbiol.

[39] Kuda T, Kawahara M, Nemoto M, Takahashi H, Kimura B (2014). *In vitro* antioxidant and anti-inflammation properties of lactic acid bacteria isolated from fish intestines and fermented fish from the Sanriku Satoumi region in Japan. Food Res Int.

[40] Yamamoto N, Shoji M, Hoshigami H, Watanabe K, Watanabe K, Takatsuzu T (2019). Antioxidant capacity of soymilk yogurt and exopolysaccharides produced by lactic acid bacteria. Biosci Microbiota Food Health.

[41] Lin X, Xia Y, Wang G, Yang Y, Xiong Z, Lv F (2018). Lactic acid bacteria with antioxidant activities alleviating oxidized oil induced hepatic injury in mice. Front Microbiol.

[42] Ilavenil S, Vijayakumar M, Kim DH, Valan Arasu M, Park HS, Ravikumar S (2016). Assessment of probiotic, antifungal and cholesterol lowering properties of *Pediococcus pentosaceus* KCC-23 isolated from Italian ryegrass. J Sci Food Agric.

[43] Kim JS, Kim JH, Palaniyandi SA, Lee CC, You JW, Yang H (2019). Yak-Kong soybean (*Glycine max*) fermented by a novel *Pediococcus pentosaceus* inhibits the oxidative stress-induced monocyte-endothelial cell adhesion. Nutrients.

[44] Lim YH, Foo HL, Loh TC, Mohamad R, Abdullah N (2019). Comparative studies of versatile extracellular proteolytic activities of lactic acid bacteria and their potential for extracellular amino acid productions as feed supplements. J Anim Sci Biotechnol.

[45] Zhao X, Higashikawa F, Noda M, Kawamura Y, Matoba Y, Kumagai T (2012). The obesity and fatty liver are reduced by plant-derived *Pediococcus pentosaceus* LP28 in high fat diet-induced obese mice. PLoS ONE.

[46] Higashikawa F, Noda M, Awaya T, Danshiitsoodol N, Matoba Y, Kumagai T (2016). Antiobesity effect of *Pediococcus pentosaceus* LP28 on overweight subjects: a randomized, double-blind, placebo-controlled clinical trial. Eur J Clin Nutr.

[47] Damodharan K, Lee YS, Palaniyandi SA, Yang SH, Suh JW (2015). Preliminary probiotic and technological characterization of *Pediococcus pentosaceus* strain KID7 and *in vivo* assessment of its cholesterol-lowering activity. Front Microbiol.

[48] Lee NY, Yoon SJ, Han DH, Gupta H, Youn GS, Shin MJ (2020). *Lactobacillus* and *Pediococcus* ameliorate progression of non-alcoholic fatty liver disease through modulation of the gut microbiome. Gut Microbes.

[49] Bengmark S (2009). Bio-ecological control of chronic liver disease and encephalopathy. Metab Brain Dis.

[50] Le B, Yang SH (2019). Biosorption of cadmium by potential probiotic *Pediococcus pentosaceus* using *in vitro* digestion model. Biotechnol Appl Biochem.

[51] Yang S, Gong P, Pan J, Wang N, Tong J, Wang M (2019). *Pediococcus pentosaceus* xy46 can absorb zearalenone and alleviate its toxicity to the reproductive systems of male mice. Microorganisms.

[52] Raghavendra P, Halami PM (2009). Screening, selection and characterization of phytic acid degrading lactic acid bacteria from chicken intestine. Int J Food Microbiol.

[53] Mohammadi-Kouchesfahani M, Hamidi-Esfahani Z, Azizi MH (2019). Isolation and identification of lactic acid bacteria with phytase activity from sourdough. Food Sci Nutr.

[54] Raghavendra P, Rao TS, Halami PM (2010). Evaluation of beneficial attributes for phytate-degrading *Pediococcus pentosaceus* CFR R123. Benef Microbes.

[55] Raghavendra P, Ushakumari SR, Halami PM (2011). Phytate-degrading *Pediococcus pentosaceus* CFR R123 for application in functional foods. Benef Microbes.

[56] Nie X, Lin S, Zhang Q (2014). Proteolytic characterisation in grass carp sausage inoculated with *Lactobacillus plantarum* and *Pediococcus pentosaceus*. Food Chem.

[57] Toe CJ, Foo HL, Loh TC, Mohamad R, Abdul Rahim R, Idrus Z (2019). Extracellular proteolytic activity and amino acid production by lactic acid bacteria isolated from Malaysian foods. Int J Mol Sci.

[58] Ratanaburee A, Kantachote D, Charernjiratrakul W, Sukhoom A (2013). Enhancement of gamma-aminobutyric acid (GABA) in Nham (Thai fermented pork sausage) using starter cultures of *Lactobacillus namurensis* NH2 and *Pediococcus pentosaceus* HN8. Int J Food Microbiol.

[59] Borges S, Teixeira P (2014). *Pediococcus pentosaceus* SB83 as a potential probiotic incorporated in a liquid system for vaginal delivery. Benef Microbes.

[60] Ghadban GS (2002). Probiotics in broiler production-a review. Arch Geflügelkd.

[61] Shani N, Oberhaensli S, Arias-Roth E (2020). Antibiotic susceptibility profiles of *Pediococcus pentosaceus* from various origins and their implications for the safety assessment of strains with food-technology applications. J Food Prot.

[62] Liang H, He Z, Wang X, Song G, Chen H, Lin X (2020). Bacterial profiles and volatile flavor compounds in commercial Suancai with varying salt concentration from Northeastern China. Food Res Int.

[63] Odutayo OE, Omonigbehin EA, Olawole TD, Ogunlana OO, Afolabi IS (2020). Fermentation enhanced biotransformation of compounds in the kernel of *Chrysophyllum albidum*. Molecules.

[64] Gong Y, Qi X (2020). A study revealing volatile aroma produced by *Pediococcus pentosaceus* in dough fermentation. Food Sci Nutr.

[65] Plessas S, Mantzourani I, Bekatorou A (2020). Evaluation of *Pediococcus pentosaceus* SP2 as starter culture on sourdough bread making. Foods.

[66] Zhang Y, Hu P, Xie Y, Wang X (2020). Co-fermentation with *Lactobacillus curvatus* LAB26 and *Pediococcus pentosaceus* SWU73571 for improving quality and safety of sour meat. Meat Sci.

[67] Bampidis V, Azimonti G, Bastos ML, Christensen H, Dusemund B, Kouba M (2020). Assessment of the application for renewal of the authorisation of *Pediococcus pentosaceus* DSM 16244 as a feed additive for all animal species. EFSA J.

[68] Yang Q, Lu Y, Zhang M, Gong Y, Li Z, Tran NT (2019). Lactic acid bacteria, *Enterococcus faecalis* Y17 and *Pediococcus pentosaceus* G11, improved growth performance, and immunity of mud crab (*Scylla paramamosain*). Fish Shellfish Immunol.

[69] Huang J, Li S, Wang Q, Guan X, Qian L, Li J (2020). *Pediococcus pentosaceus* B49 from human colostrum ameliorates constipation in mice. Food Funct.

[70] Kuppusamy P, Kim D, Soundharrajan I, Park HS, Jung JS, Yang SH (2020). Low-carbohydrate tolerant LAB strains identified from rumen fluid: Investigation of probiotic activity and legume silage fermentation. Microorganisms.

[71] Eveno M, Savard P, Belguesmia Y, Bazinet L, Gancel F, Drider D (2020). Compatibility, cytotoxicity, and gastrointestinal tenacity of bacteriocin-producing bacteria selected for a consortium probiotic formulation to be used in livestock feed. Probiotics Antimicrob Proteins.

[72] Vadopalas L, Ruzauskas M, Lele V, Starkute V, Zavistanaviciute P, Zokaityte E (2020). Combination of antimicrobial starters for feed fermentation: influence on piglet feces microbiota and health and growth performance, including mycotoxin biotransformation *in vivo*. Front Vet Sci.

[73] Meinen A, Simon S, Banerji S, Szabo I, Malorny B, Borowiak M (2019). Salmonellosis outbreak with novel *Salmonella enterica* subspecies *enterica* serotype (11:z41:e, n, z15) attributable to sesame products in five European countries, 2016 to 2017. Eurosurveillance.

[74] Nze UC, Beeman MG, Lambert CJ, Salih G, Gale BK, Sant HJ (2019). Hydrodynamic cavitation for the rapid separation and electrochemical detection of *Cryptosporidium parvum* and *Escherichia coli* O157:H7 in ground beef. Biosens Bioelectron.

[75] Maury MM, Bracq-Dieye H, Huang L, Vales G, Lavina M, Thouvenot P (2019). Hypervirulent *Listeria monocytogenes* clones' adaption to mammalian gut accounts for their association with dairy products. Nat Commun.

[76] Orsi RH, Wiedmann M (2016). Characteristics and distribution of *Listeria* spp. including *Listeria* species newly described since 2009. Appl Microbiol Biotechnol..

[77] Lecuit M (2020). *Listeria monocytogenes*, a model in infection biology. Cell Microbiol.

[78] Ladha G, Jeevaratnam K (2020). Characterization of purified antimicrobial peptide produced by *Pediococcus pentosaceus* LJR1, and its application in preservation of white leg shrimp. World J Microbiol Biotechnol.

[79] De Azevedo POS, Mendonca CMN, Seibert L, Dominguez JM, Converti A, Gierus M (2020). Bacteriocin-like inhibitory substance of *Pediococcus pentosaceus* as a biopreservative for *Listeria* sp. control in ready-to-eat pork ham. Braz J Microbiol..

[80] Nanasombat S, Treebavonkusol P, Kittisrisopit S, Jaichalad T, Phunpruch S, Kootmas A (2017). Lactic acid bacteria isolated from raw and fermented pork products: identification and characterization of catalase-producing *Pediococcus pentosaceus*. Food Sci Biotechnol.

[81] Ben Taheur F, Kouidhi B, Fdhila K, Elabed H, Ben Slama R, Mahdouani K (2016). Anti-bacterial and anti-biofilm activity of probiotic bacteria against oral pathogens. Microb Pathog.

[82] Yazgan H, Kuley E, Guven Gokmen T, Regenstein JM, Ozogul F (2020). The antimicrobial properties and biogenic amine production of lactic acid bacteria isolated from various fermented food products. J Food Process Preserv.

[83] Diguța CF, Nițoi GD, Matei F, Luța G, Cornea CP (2020). The Biotechnological potential of *Pediococcus* spp. isolated from kombucha microbial consortium. Foods (Basel, Switzerland)..

[84] Verni M, Wang C, Montemurro M, De Angelis M, Katina K, Rizzello CG (2017). Exploring the microbiota of faba bean: functional characterization of lactic acid bacteria. Front Microbiol.

[85] Magnusson J, Ström K, Roos S, Sjögren J, Schnürer J (2003). Broad and complex antifungal activity among environmental isolates of lactic acid bacteria. FEMS Microbiol Lett.

[86] Varsha KK, Priya S, Devendra L, Nampoothiri KM (2014). Control of spoilage fungi by protective lactic acid bacteria displaying probiotic properties. Appl Biochem Biotechnol.

[87] Bartkiene E, Lele V, Ruzauskas M, Domig KJ, Starkute V, Zavistanaviciute P (2020). Lactic acid bacteria isolation from spontaneous sourdough and their characterization including antimicrobial and antifungal properties evaluation. Microorganisms.

[88] Sellamani M, Kalagatur NK, Siddaiah C, Mudili V, Krishna K, Natarajan G (2016). Antifungal and zearalenone inhibitory activity of *Pediococcus pentosaceus* isolated from dairy products on *Fusarium graminearum*. Front Microbiol.

[89] Bulgasem BY, Lani MN, Hassan Z, Yusoff WMW, Fnaish SG (2016). Antifungal activity of lactic acid bacteria strains isolated from natural honey against pathogenic candida species. Mycobiology.

[90] Dalie DK, Deschamps AM, Atanasova-Penichon V, Richard-Forget F (2010). Potential of *Pediococcus pentosaceus* (L006) isolated from maize leaf to suppress fumonisin-producing fungal growth. J Food Prot.

[91] Shin JM, Gwak JW, Kamarajan P, Fenno JC, Rickard AH, Kapila YL (2016). Biomedical applications of nisin. J Appl Microbiol.

[92] De Azevedo POS, Converti A, Dominguez JM, Oliveira RPS (2017). Stimulating effects of sucrose and inulin on growth, lactate, and bacteriocin productions by *Pediococcus pentosaceus*. Probiotics Antimicrob Proteins.

[93] Porto MC, Kuniyoshi TM, Azevedo PO, Vitolo M, Oliveira RP (2017). Pediococcus spp.: an important genus of lactic acid bacteria and pediocin producers. Biotechnol Adv..

[94] Ghosh B, Sukumar G, Ghosh AR (2019). Purification and characterization of pediocin from probiotic *Pediococcus pentosaceus* GS4, MTCC 12683. Folia Microbiol (Praha).

[95] Shin MS, Han SK, Ji AR, Kim KS, Lee WK (2008). Isolation and characterization of bacteriocin-producing bacteria from the gastrointestinal tract of broiler chickens for probiotic use. J Appl Microbiol.

[96] Shin MS, Han SK, Ryu JS, Kim KS, Lee WK (2008). Isolation and partial characterization of a bacteriocin produced by *Pediococcus pentosaceus* K23–2 isolated from Kimchi. J Appl Microbiol.

[97] Bungenstock L, Abdulmawjood A, Reich F (2020). Evaluation of antibacterial properties of lactic acid bacteria from traditionally and industrially produced fermented sausages from Germany. PLoS ONE.

[98] Pinto A, Barbosa J, Albano H, Isidro J, Teixeira P (2020). Screening of bacteriocinogenic lactic acid bacteria and their characterization as potential probiotics. Microorganisms.

[99] Soundharrajan I, Kim D, Kuppusamy P, Muthusamy K, Lee HJ, Choi KC (2019). Probiotic and *Triticale* silage fermentation potential of *Pediococcus pentosaceus* and *Lactobacillus brevis* and their impacts on pathogenic bacteria. Microorganisms.

[100] Cavicchioli VQ, Camargo AC, Todorov SD, Nero LA (2017). Novel bacteriocinogenic *Enterococcus hirae* and *Pediococcus pentosaceus* strains with antilisterial activity isolated from Brazilian artisanal cheese. J Dairy Sci.

[101] Cavicchioli VQ, Camargo AC, Todorov SD, Nero LA (2019). Potential control of *Listeria monocytogenes* by bacteriocinogenic *Enterococcus hirae* ST57ACC and *Pediococcus pentosaceus* ST65ACC strains isolated from artisanal cheese. Probiotics Antimicrob Proteins.

[102] Todorov SD, Cavicchioli VQ, Ananieva M, Bivolarski VP, Vasileva TA, Hinkov AV (2020). Expression of coagulin A with low cytotoxic activity by *Pediococcus pentosaceus* ST65ACC isolated from raw milk cheese. J Appl Microbiol.

[103] Vidhyasagar V, Jeevaratnam K (2013). Bacteriocin activity against various pathogens produced by *Pediococcus pentosaceus* VJ13 isolated from Idly batter. Biomed Chromatogr.

[104] Osmanagaoglu O, Kiran F, Ataoglu H (2010). Evaluation of *in vitro* probiotic potential of *Pediococcus pentosaceus* OZF isolated from human breast milk. Probiotics Antimicrob Proteins.

[105] Todorov SD, Dicks LM (2009). Bacteriocin production by *Pediococcus pentosaceus* isolated from marula (*Scerocarya birrea*). Int J Food Microbiol.

[106] Caldwell SL, McMahon DJ, Oberg CJ, Broadbent JR (1996). Development and characterization of lactose-positive *Pediococcus* species for milk fermentation. Appl Environ Microbiol.

[107] Snauwaert I, Stragier P, De Vuyst L, Vandamme P (2015). Comparative genome analysis of Pediococcus damnosus LMG 28219, a strain well-adapted to the beer environment. BMC Genomics.

[108] Diep DB, Godager L, Brede D, Nes IF (2006). Data mining and characterization of a novel pediocin-like bacteriocin system from the genome of *Pediococcus pentosaceus* ATCC 25745. Microbiology (Reading).

[109] Yin LJ, Wu CW, Jiang ST (2003). Bacteriocins from *Pediococcus pentosaceus* L and S from pork meat. J Agric Food Chem.

[110] Zommiti M, Bouffartigues E, Maillot O, Barreau M, Szunerits S, Sebei K (2018). *In vitro* assessment of the probiotic properties and bacteriocinogenic potential of *Pediococcus pentosaceus* MZF16 isolated from artisanal Tunisian meat “Dried Ossban”. Front Microbiol.

[111] Kaur R, Tiwari SK (2018). Membrane-acting bacteriocin purified from a soil isolate *Pediococcus pentosaceus* LB44 shows broad host-range. Biochem Biophys Res Commun.

[112] Miller KW, Ray P, Steinmetz T, Hanekamp T, Ray B (2005). Gene organization and sequences of pediocin AcH/PA-1 production operons in *Pediococcus* and *Lactobacillus* plasmids. Lett Appl Microbiol.

[113] Wu CW, Yin LJ, Jiang ST (2004). Purification and characterization of bacteriocin from *Pediococcus pentosaceus* ACCEL. J Agric Food Chem.

[114] Piva A, Meola E, Panciroli A (1995). Effect of *Pediococcus pentosaceus* FBB61, pediocin A producer strain, in caecal fermentations. J Appl Bacteriol.

[115] de Nadra MCM, de Lamelas DS, de Saad AMS (1998). Pediocin N5p from *Pediococcus pentosaceus*: adsorption on bacterial strains. Int J Food Microbiol.

[116] Costilow RN, Coughlin FM, Robach DL, Ragheb HS (1956). A study of the acid-forming bacteria from cucumber fermentations in Michigan. J Food Sci.

[117] De Azevedo POS, De Azevedo HF, Figueroa E, Converti A, Dominguez JM, Oliveira RPS (2019). Effects of pH and sugar supplements on bacteriocin-like inhibitory substance production by *Pediococcus pentosaceus*. Mol Biol Rep.

[118] De Azevedo POS, Converti A, Gierus M, Oliveira RPS (2019). Antimicrobial activity of bacteriocin-like inhibitory substance produced by *Pediococcus pentosaceus*: from shake flasks to bioreactor. Mol Biol Rep.

[119] Gutierrez-Cortes C, Suarez H, Buitrago G, Nero LA, Todorov SD (2018). Enhanced bacteriocin production by *Pediococcus pentosaceus* 147 in co-culture with *Lactobacillus plantarum* LE27 on cheese whey broth. Front Microbiol.

[120] Merenstein DJ, Sanders ME, Tancredi DJ (2020). Probiotics as a Tx resource in primary care. J Fam Pract.

[121] Degnan FH (2008). The US food and drug administration and probiotics: regulatory categorization. Clin Infect Dis..

[122] Koutsoumanis K, Allende A, Alvarez-Ordonez A, Bolton D, Bover-Cid S, Chemaly M (2020). Update of the list of QPS-recommended biological agents intentionally added to food or feed as notified to EFSA 12: suitability of taxonomic units notified to EFSA until March 2020. EFSA J.

[123] Dubey V, Mishra AK, Ghosh AR (2020). Cell adherence efficacy of probiotic *Pediococcus pentosaceus* GS4 (MTCC 12683) and demonstrable role of its surface layer protein (Slp). J Proteomics.

[124] Weckx S, Van der Meulen R, Allemeersch J, Huys G, Vandamme P, Van Hummelen P (2010). Community dynamics of bacteria in sourdough fermentations as revealed by their metatranscriptome. Appl Environ Microbiol.

[125] Pradhan P, Tamang JP (2019). Phenotypic and genotypic identification of bacteria isolated from traditionally prepared dry starters of the Eastern Himalayas. Front Microbiol.

[126] Ha JH, Kim AR, Lee KS, Xuan SH, Kang HC, Lee DH (2019). Anti-aging activity of *Lavandula angustifolia* extract fermented with *Pediococcus pentosaceus* DK1 isolated from *Diospyros kaki* fruit in UVB-irradiated human skin fibroblasts and analysis of principal components. J Microbiol Biotechnol.

[127] Chen Q, Kong B, Sun Q, Dong F, Liu Q (2015). Antioxidant potential of a unique LAB culture isolated from Harbin dry sausage: in vitro and in a sausage model. Meat Sci.

[128] Wang Y, You Y, Tian Y, Sun H, Li X, Wang X (2020). *Pediococcus pentosaceus* PP04 ameliorates high-fat diet-induced hyperlipidemia by regulating lipid metabolism in C57BL/6N mice. J Agric Food Chem.

[129] Patel S, Kasoju N, Bora U, Goyal A (2010). Structural analysis and biomedical applications of dextran produced by a new isolate *Pediococcus pentosaceus* screened from biodiversity hot spot Assam. Bioresour Technol.

[130] Duchaine C, Israel-Assayag E, Fournier M, Cormier Y (1996). Proinflammatory effect of *Pediococcus pentosaceus*, a bacterium used as hay preservative. Eur Respir J.

[131] Suez J, Zmora N, Segal E, Elinav E (2019). The pros, cons, and many unknowns of probiotics. Nat Med.

[132] Wang Y, Jiang Y, Deng Y, Yi C, Wang Y, Ding M (2020). Probiotic supplements: hope or hype?. Front Microbiol.

[133] Sukumar G, Ghosh AR (2010). *Pediococcus* spp.—a potential probiotic isolated from khadi (an Indian fermented food) and identified by 16S rDNA sequence analysis. Afr J Food Sci..

[134] Dubey V, Ghosh AR, Mandal BK (2012). Appraisal of conjugated linoleic acid production by probiotic potential of *Pediococcus* spp. GS4. Appl Biochem Biotechnol..

[135] Dubey V, Ghosh AR, Bishayee K, Khuda-Bukhsh AR (2015). Probiotic *Pediococcus pentosaceus* strain GS4 alleviates azoxymethane-induced toxicity in mice. Nutr Res.

[136] Dubey V, Ghosh AR, Bishayee K, Khuda-Bukhsh AR (2016). Appraisal of the anti-cancer potential of probiotic *Pediococcus pentosaceus* GS4 against colon cancer: in vitro and in vivo approaches. J Funct Foods.

[137] Bagad M, Pande R, Dubey V, Ghosh AR (2017). Survivability of freeze-dried probiotic *Pediococcus pentosaceus* strains GS4, GS17 and *Lactobacillus gasseri* (ATCC 19992) during storage with commonly used pharmaceutical excipients within a period of 120 days. Asian Pac J Trop Biomed.

[138] An KS, Choi YO, Lee SM, Ryu HY, Kang SJ, Yeon Y (2019). Ginsenosides Rg5 and Rk1 enriched cultured wild ginseng root extract bioconversion of *Pediococcus pentosaceus* HLJG0702: effect on scopolamine-induced memory dysfunction in mice. Nutrients.

[139] Lv LX, Hu XJ, Qian GR, Zhang H, Lu HF, Zheng BW (2014). Administration of *Lactobacillus salivarius* LI01 or *Pediococcus pentosaceus* LI05 improves acute liver injury induced by D-galactosamine in rats. Appl Microbiol Biotechnol.

[140] Shi D, Lv L, Fang D, Wu W, Hu C, Xu L (2017). Administration of *Lactobacillus salivarius* LI01 or *Pediococcus pentosaceus* LI05 prevents CCl4-induced liver cirrhosis by protecting the intestinal barrier in rats. Sci Rep.

[141] Xu Q, Gu S, Chen Y, Quan J, Lv L, Chen D (2018). Protective effect of *Pediococcus pentosaceus* LI05 against clostridium difficile infection in a mouse model. Front Microbiol.

[142] Bian X, Yang L, Wu W, Lv L, Jiang X, Wang Q (2020). *Pediococcus pentosaceus* LI05 alleviates DSS-induced colitis by modulating immunological profiles, the gut microbiota and short-chain fatty acid levels in a mouse model. Microb Biotechnol.

[143] Bove P, Gallone A, Russo P, Capozzi V, Albenzio M, Spano G (2012). Probiotic features of *Lactobacillus plantarum* mutant strains. Appl Microbiol Biotechnol.

[144] Lee KW, Park JY, Sa HD, Jeong JH, Jin DE, Heo HJ (2014). Probiotic properties of Pediococcus strains isolated from jeotgals, salted and fermented Korean sea-food. Anaerobe.

[145] Todhanakasem T, Triwattana K, Pom J, Havanapan P, Koombhongse P, Thitisak P (2020). Physiological studies of the *Pediococcus pentosaceus* biofilm. Lett Appl Microbiol.

